# Tissue Plasminogen Activator Causes Brain Microvascular Endothelial Cell Injury After Oxygen Glucose Deprivation by Inhibiting Sonic Hedgehog Signaling

**DOI:** 10.1007/s11064-018-2697-2

**Published:** 2018-12-14

**Authors:** Pian Gong, Mingchang Li, Changlin Zou, Qi Tian, Zhou Xu

**Affiliations:** Department of Neurosurgery, Renmin Hospital of Wuhan University, No. 9 Zhangzhidong Road, Wuchang District, Wuhan, 430072 Hubei Province China

**Keywords:** Tissue plasminogen activator, Sonic hedgehog signaling pathway, Brain microvascular endothelial cells, Oxygen and glucose deprivation, Blood–brain barrier

## Abstract

The thrombolytic activity of tissue plasminogen activator (tPA) has undisputed benefits. However, the documented neurotoxicity of tPA raises important issues. Currently, common treatments for stroke might not be optimum if exogenous tPA can pass through the blood–brain barrier and enter the brain, thus adding to the deleterious effects of tPA within the cerebral parenchyma. Here, we determined whether tPA could damage brain microvascular endothelial cells (BMECs) during cerebral ischemia. We showed that treatment of BMECs with tPA decreased trans-endothelial electrical resistance and cell proliferation, and blocked the cell cycle at the G0–G1 phase. In addition, the Sonic hedgehog (Shh) signaling pathway was involved in tPA-induced BMECs dysfunction. However, tPA-enhanced oxygen glucose deprivation-induced BMECs dysfunction was eliminated by Shh administration and the effects could be reversed by Shh inhibitors. Taken together, these results demonstrate that tPA administration might result in damage to the endothelial barrier owing to blocked Shh signaling pathway.

## Introduction

Stroke leads to the disruption of the blood–brain barrier (BBB), which increases the permeability of the brain microvasculature and eventually results in brain edema [1]. The BBB is formed by brain endothelial cells lining cerebral microvessels and performs a combination of physical, transport, and enzymatic barrier functions [2]. Endothelial cells have the capacity to modulate barrier permeability by regulating the expression of tight and adherent junction proteins. Therefore, endothelial cell injury can influence the permeability of the BBB [3, 4]. Brain microvascular endothelial cells (BMECs) play an important role in maintaining brain vascular homeostasis, and dysfunction of the endothelial layer is recognized as an early event in the pathogenesis of cerebrovascular diseases such as subarachnoid hemorrhage and ischemic stroke [5, 6].

Tissue plasminogen activator (tPA) is a serine protease that induces the conversion of plasminogen to plasmin to dissolve fibrin-based blood clots and is routinely used in ischemic stroke pharmacotherapy [7, 8]. However, treatment via tPA thrombolysis is limited to less than 5% of stroke patients due to the narrow therapeutic time window and the potentially devastating complication of intracerebral hemorrhage [9, 10]. tPA-related intracerebral hemorrhage occurs as a consequence of severe BBB disruption during thrombolytic reperfusion [11]. Previously, in vitro studies using BMECs and astrocytes have shown that endothelial tPA expression is negatively regulated by astrocytes [12]. A growing body of evidence suggests that exacerbation of BBB injury following tPA administration, such as the induction of proteolytic degradation of the structural components of the BBB, increases the production of free radicals and induces toxicity to neurovascular cells [13–15]. However, the molecular effects associated with tPA administration after ischemic stroke remain unknown.

Sonic hedgehog (Shh) is a glycoprotein that has both morphogenic and mitogenic properties and is an indirect angiogenic factor in individual development and tissue repairment [16]. Shh binds to the specific receptor Patched-1 (Ptch1), thereby causing the release of the transmembrane protein Smoothened (Smo). This leads to the activation of the transcription factor Gli-1, which induces the expression of genes in downstream signaling pathways and regulates endothelial cell apoptosis [17, 18]. It has also been suggested that Shh induces cerebral angiogenesis and mediates neural-tube angiogenesis during embryonic development [19, 20]. In addition, Shh promotes the integrity and immune quiescence of the BBB by decreasing the expression of inflammatory mediators as well as the adhesion and migration of leucocytes [21].

In the present study, we investigated the effect of tPA administration on the Shh signal pathway in BMECs. This study was primarily designed to elucidate the role of Shh in the effects of tPA administration after ischemic stroke.

## Materials and Methods

### Ethic Statements

This study was approved by the committees on experimental animal ethics of Renmin Hospital, Wuhan University. All animal experiments were performed in accordance with the Guidelines for Experimental Animals of the Ministry of Science and Technology (Beijing, China). All dissections were performed according to recommendations of the European Commission, and all efforts were made to minimize suffering in our animals.

### Isolation of Rat BMECs

BMECs were isolated from Sprague–Dawley rats, according to reported protocols with some modifications [22]. Briefly, rats were sacrificed and their brains were collected. Subsequently, white matter, brain stems, surface vessels, and leptomeninges were removed carefully. The isolated cerebral cortices were placed in ice-cold phosphate-buffered saline (PBS), minced into small pieces, and homogenized. The homogenates were centrifuged at 500×*g* for 5 min at 4 °C. The pellet was resuspended in 20% bovine serum albumin (BSA) and centrifuged at 1000×*g* for 20 min at 4 °C. The microvessels in the lower layer were transferred to a new tube. After one PBS wash, the microvessel pellets were digested using 0.1% collagenase II/dispase and 1000 U/ml DNase I at 37 °C for 1 h. Following this, the microvessel pellets were centrifuged at 500×*g* for 5 min at 4 °C and resuspended in 10 ml of M131 medium (Invitrogen, USA) supplemented with microvascular growth supplement (Invitrogen, USA), 100 U/ml penicillin, and 100 U/ml streptomycin. The cell suspension was seeded into a 75-cm^2^ flask and incubated at 37 °C in humidified 5% CO_2_/95% air. BMECs at passages 3 to 6 were used.

### Oxygen Glucose Deprivation and Drug Treatment

In order to simulate ischemic conditions, oxygen glucose deprivation (OGD) was induced in the *in vitro* cell study. Cells were exposed to OGD medium with low glucose (0.2 g/l) equilibrated with nitrogen (Gibco, USA). Hypoxic conditions were achieved using a chamber with 95% N_2_ and 5% CO_2_ at 37 °C for 4 h. PBS (control group, CON), tPA (300 µg/ml), Shh (3 µg/ml), or cyclopamine (20 µM) were added after the cells were subjected to OGD for 4 h, following which they were cultured under normoxic conditions for another 24 h and harvested for different experiments [23]. Each experiment was performed in triplicate.

### Immunofluorescence

For immunofluorescence, cells were seeded onto 1.5-mm glass coverslips coated with 0.2% gelatin. The cells were washed with PBS and fixed in 4% paraformaldehyde for 15 min, followed by permeabilization in 0.2% Triton X-100 for 10 min. After being blocked in 5% BSA for 1 h at room temperature, the cells were incubated with primary antibodies (VIII factor or CD34) for 1 h at room temperature. The cells were washed and incubated for 1 h with Alexa Fluor-conjugated secondary antibodies and 4′-6-diamidino-2-phenylindole as a nuclear counterstain. The coverslips were washed and imaged using a ZeissAxioplan II microscope (Carl Zeiss, USA).

### Measurement of Trans-endothelial Electrical Resistance (TEER)

The integrity of the BMECs was evaluated by measuring the TEER using a Millicell-ERS device (Millipore, Bedford, USA). Cells were allowed to grow until a TEER value of greater than 1000 Ω cm^2^ was reached, indicating the formation of a tight epithelial monolayer [24]. TEER measurements were made using an epithelial volt-ohm-meter (EVOM, World Precision Instruments Inc., Sarasota, USA). Changes in TEER were expressed as the percentage of the initial value adjusted for control cell layers according to the equation:

$${\text{TEER}}\left( \% \right)=\left( {{{{\text{final}}\;{\text{TEE}}{{\text{R}}_{{\text{treated}}}}} \mathord{\left/ {\vphantom {{{\text{final}}\;{\text{TEE}}{{\text{R}}_{{\text{treated}}}}} {{\text{final}}\;{\text{TEE}}{{\text{R}}_{{\text{control}}}}}}} \right. \kern-0pt} {{\text{final}}\;{\text{TEE}}{{\text{R}}_{{\text{control}}}}}}} \right) \times \left( {{{{\text{initial}}\;{\text{TEE}}{{\text{R}}_{{\text{control}}}}} \mathord{\left/ {\vphantom {{{\text{initial}}\;{\text{TEE}}{{\text{R}}_{{\text{control}}}}} {{\text{initial}}\;{\text{TEE}}{{\text{R}}_{{\text{treated}}}}}}} \right. \kern-0pt} {{\text{initial}}\;{\text{TEE}}{{\text{R}}_{{\text{treated}}}}}}} \right) \times 100$$

### Proliferation Assay

The cell-counting kit 8 assay (CCK-8) was used to evaluate cell proliferation. Cells were seeded in 96-well plates with 1 × 10^4^ cells in each well. Subsequently, 10 µl of CCK-8 reagent (5 mg/ml) was added to each well, and the plates were incubated at 37 °C for 2 h. The optical density was measured at 450 nm.

### Cell Cycle Assay

The cell cycle of rat BMECs was examined using Lipofectamine 2000. Cells were washed thrice with ice-cold PBS and fixed with 70% (v/v) ethanol at − 70 °C for 1 h. Following the PBS wash, a staining solution containing 10 mM Tris (pH 7.0), 0.1% NP-40, 1 mM NaCl, 0.7 µg/ml ribonuclease A, and 5 µg/ml propidium iodide (PI) was added to the cells. After incubation for 30 min in the dark, the cellular DNA content was examined by PI-staining flow cytometry.

### RNA Extraction and Quantitative Reverse-Transcription Polymerase Chain Reaction (qRT-PCR)

Total RNA was extracted from samples with Trizol reagent (TaKaRa, Dalian, China) and detected by an ultraviolet spectrophotometer and agarose electrophoresis. For each sample, 1 µg of total RNA was reverse-transcribed to obtain first-strand cDNA using the PrimeScript®RT reagent Kit with gDNA Eraser (TaKaRa, Dalian, China) according to the manufacturer’s instructions. Primer Premier 5.0 was used to design the fluorescent primers before gene synthesis. The synopsis of primers for each gene is listed in Table 1. The reaction mixture (total volume of 20 µl) contained 10 µl of 2 × SYBR Premix Ex TaqTM (TaKaRa, Dalian, China), 0.4 µl of each primer, and 0.2 ± 0.02 µl of cDNA template. The following qRT-PCR reaction was performed: pre-denaturation at 95 °C for 30 s, followed by 40 cycles of denaturation at 95 °C for 3 min, annealing at 56–60 °C for 20 s, and elongation at 72 °C for 20 s. The transcriptional levels of genes were calculated using the ^ΔΔ^Ct method. The threshold cycle (Ct) was determined for each reaction using the ^ΔΔ^Ct method, and each gene of interest was normalized to the endogenous control gene (GAPDH). For each group, three samples were measured and three technical replicates of each measurement were obtained.

**Table 1:** Primers used for qRT-PCR analysis

Primer	Forward primer (5′-3′)	Reverse primer (5′-3′)	TA (°C)
GAPDH	CCTTCCGTGTTCCTAC	GACAACCTGGTCCTCA	60
Shh	AGCCTACAAGCAGTTTATT	TCTTTGCACCTCTGAGTC	56
Ptch1	AATCAGGGGAACTTATCA	ACCGTAAAGGAGGCTTA	56
Smo	CTGACTTTCTGCGTTGC	TTGGGGTTGTCTGTTCG	58
Gli-1	GGATACAACCCAAATGC	TGGCGAATAGACAGAGG	56
Gli-2	TGACCGAAGTGACGATG	AAGTATGGGGAGATGCC	56
Gli-3	CCCCTACATCAACCCAT	CCTGTCAGCAGAGCCAT	58
ZO-1	TGCTCCAGCAGGTCCTAAGT	TGGTAGCTGAGGGCAGAACT	58
Claudin-3	CATCCTGCTGGCCGCCTTCG	CCTGATGATGGTGTTGGCCGAC	56

### Western Blot

Protein expression levels were analyzed by western blot. For total protein extraction, cells were washed twice with PBS and lysed at 4 °C with RIPA buffer (Beyotime, China) containing protease inhibitor. The cell lysate was centrifuged at 12,000×*g* for 15 min and the supernatants were collected. The protein concentration was determined by a bicinchoninic acid kit (Bioswamp, China). Equal amounts of protein (30 µg) were separated by 10% sodium dodecyl sulfate–polyacrylamide electrophoresis, after which the proteins were transferred onto polyvinylidene fluoride membranes (Millipore, USA) at 200 mA for 2 h. The membranes were blocked for 2 h at room temperature with 5% skim milk in Tris-buffered saline (20 mM Tris, 500 mM NaCl, and 0.05% Tween 20). Subsequently, the membranes were incubated with primary antibodies against Shh (ab19897, 1:1000, abcam), Ptch1 (ab53715, 1:1000, abcam), Smo (ab72130, 1:1000, abcam), Gli-1 (ab49314, 1:500, abcam), Gli-2 (ab167398, 1:1000, abcam), Gil-3 (ab69838, 1:1000, abcam), ZO-1 (Sc-8146, 1:500, Santa-Cruz Biotechnology), and claudin-3 (ab116165, 1:1000, abcam) overnight at 4 °C. GAPDH (2118, 1:10000, CST) was selected as the internal reference. After primary antibody incubation, the membranes were washed with Tris-buffered saline and incubated in biotinylated goat IgG secondary antibody (PAB160011, 1:10000, Bioswamp) for 2 h at room temperature. Immunoreactivity was visualized by colorimetric reaction using an enhanced chemiluminescence substrate buffer (Millipore, Massachusetts, USA). The membranes were scanned with the Gel Doz EZ imager (Bio-Rad, USA).

### Statistical Analysis

Statistical significance of differences between the experimental data were evaluated via one-way analysis of variance (ANOVA), using the SPSS 19.0 software package. Differences were considered statistically significant at P < 0.05. All results are expressed as mean ± standard deviation (SD).

## Results

### Observation and Identification of Rat BMECs

**Fig. 1:**
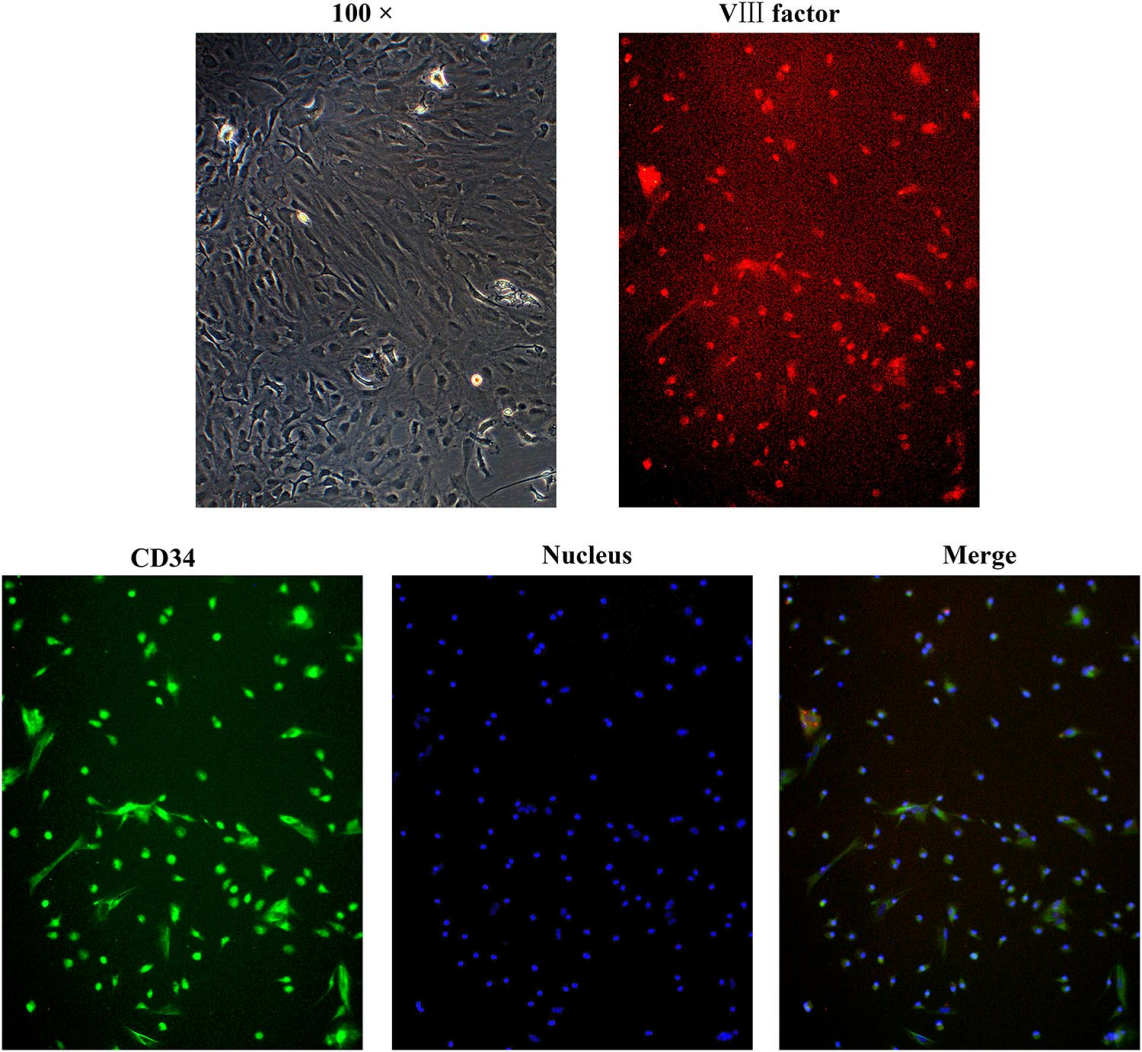
BMECs were isolated and identified by optical and fluorescence microscopy (100 ×). Cells formed a confluent monolayer and exhibited a cobblestone pattern. Immunofluorescence showed that the cells were positively stained for VIII factor and CD34, suggesting the presence of BMECs

Rat BMECs were successfully isolated from brain tissue. Cells formed a confluent monolayer and exhibited a cobblestone pattern (Fig. 1a). Immunofluorescence showed that the cells were positively stained for VIII factor (Fig. 1b) and CD34 (Fig. 1c), suggesting that BMECs were present.

### tPA Decreased TEER of BMECs

**Fig. 2:**
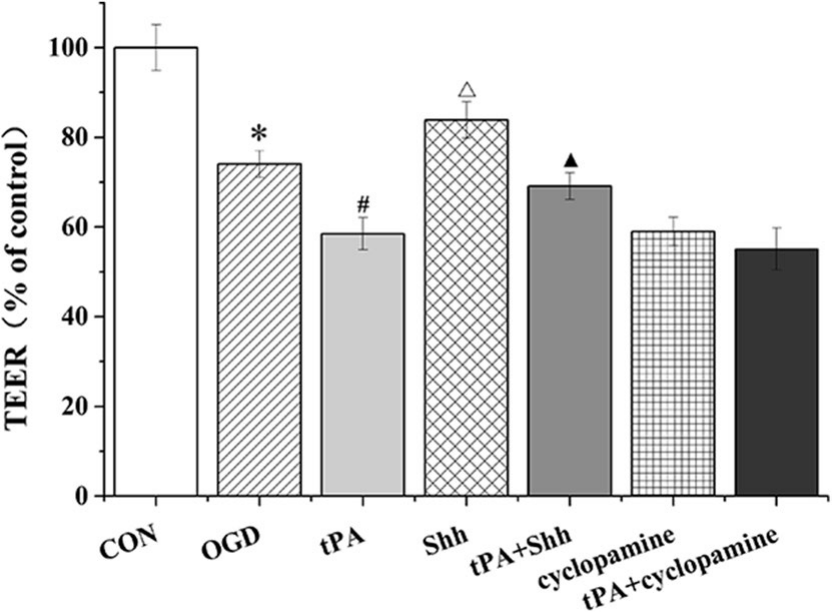
The TEER of BMECs was detected by a Millicell-ERS device. Data are shown as mean ± SD (n = 3); *P < 0.05 versus CON group, ^#^P < 0.05 versus OGD group, ^△^P < 0.05 versus tPA group, ^▲^P < 0.05 versus Shh group

The TEER of BMECs was detected by a Millicell-ERS device (Fig. 2). OGD and tPA administration significantly decreased the TEER of rat BMECs. However, Shh significantly inhibited the OGD- or tPA-induced decrease in TEER.

### tPA Inhibited the Proliferation of BMECs

**Fig. 3:**
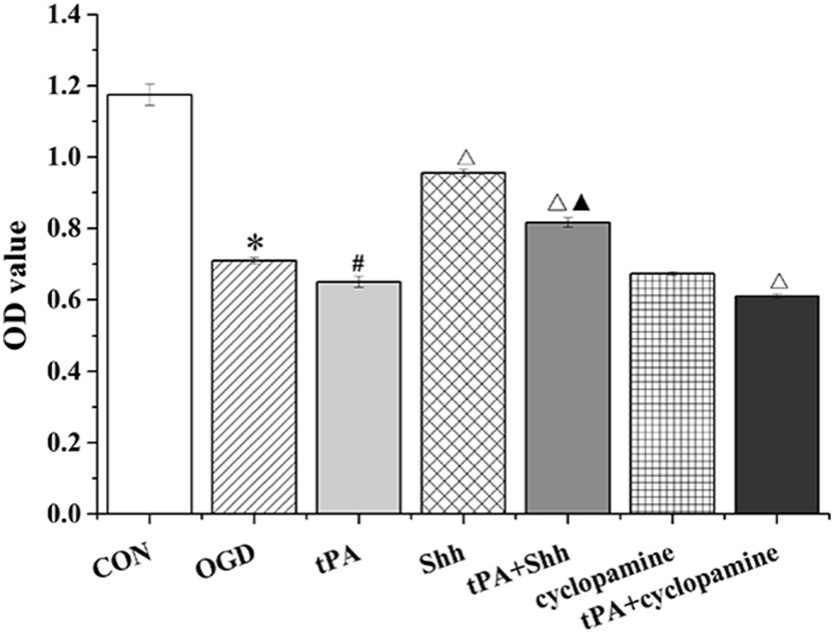
The proliferation of BMECs was detected by CCK-8 assay. Data are shown as mean ± SD (n = 3); *P < 0.05 versus CON group, ^#^P < 0.05 versus OGD group, ^△^P < 0.05 versus tPA group, ^▲^P < 0.05 versus Shh group

To explore the viability of BMECs following tPA administration, a CCK-8 assay was performed to evaluate the cytotoxicity of tPA toward cells. We found that the proliferation of BMECs was significantly inhibited by OGD or tPA. However, tPA administration combined with Shh treatment significantly inhibited the tPA-induced decrease in BMEC proliferation. Conversely, after treatment with cyclopamine, cell proliferation decreased significantly (Fig. 3).

### tPA Affected the Cell Cycle of BMECs

**Fig. 4:**
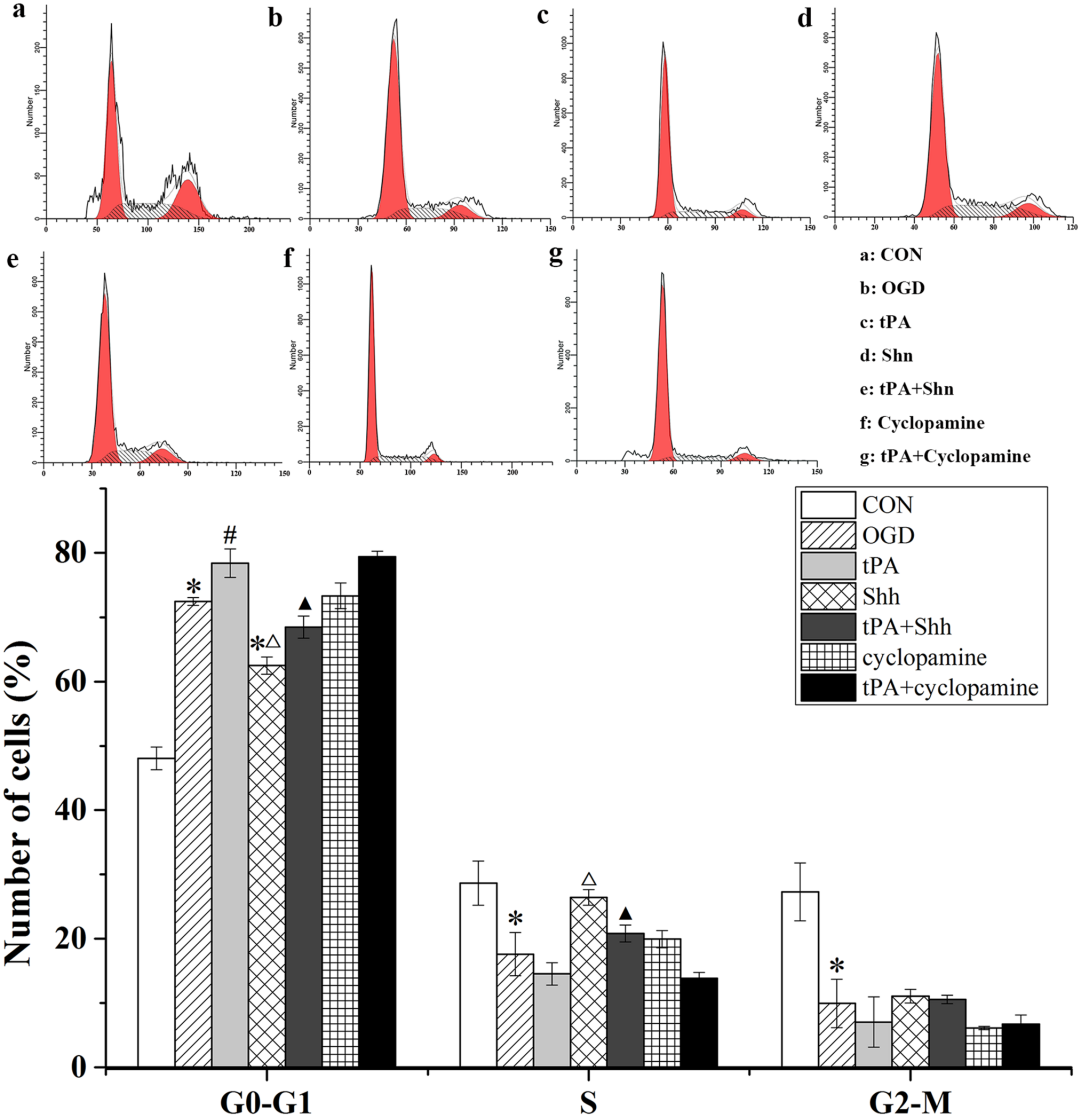
tPA induced cell cycle arrest in G0–G1 phase. The cell cycle distribution of BMECs was measured by flow cytometry. Representative histograms and the cell number in each cell cycle are plotted as mean ± SD (n = 3). *P < 0.05 versus CON group, ^#^P < 0.05 versus OGD group, ^△^P < 0.05 versus tPA group, ^▲^P < 0.05 versus Shh group

Because we observed that tPA inhibited cell proliferation in BMECs, we investigated the effect of tPA on the cell cycle of BMECs. As shown in Fig. 4, significant G0–G1 cell cycle arrest was detected in BMECs treated with OGD or tPA, accompanied by a reduction in cell number in the S-phase. Shh repaired the arrest and sped up the cell process. Unfortunately, the recovery function of Shh could be offset by tPA administration. These results suggest that Shh is involved in OGD- or tPA-induced BMEC cycle arrest in the G0-G1 phase.

### tPA Blocked the Shh Signaling Pathway in BMECs and Upregulated the Expression of ZO-1 and Claudin-3

**Fig. 5:**
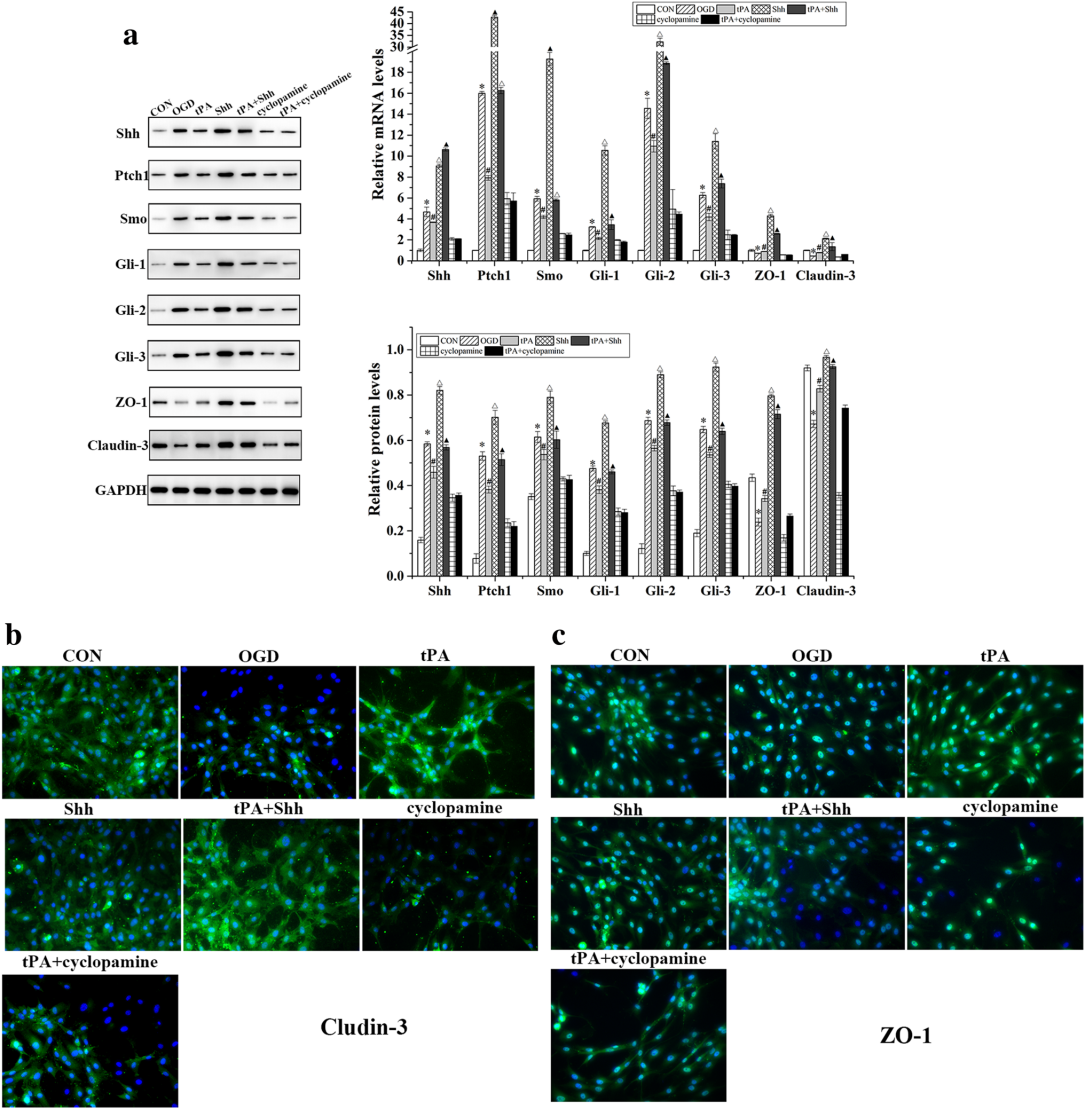
**a** Western blot and qRT-PCR analysis of Shh, Smo, Ptch1, Gli-1, Gli-2, Gli-3, ZO-1, and claudin-3 expression in BMECs. Bands were quantified using Quantity One 5.0. Claudin-3 (**b**) and ZO-1 (**c**) expressions were detected by immunofluorescence assay in BMECs (200 × amplification). Data are shown as mean ± SD (n = 3); *P < 0.05 versus CON group, ^#^P < 0.05 versus OGD group, ^△^P < 0.05 versus tPA group, ^▲^P < 0.05 versus Shh group

Western blot and qRT-PCR were performed to evaluate the effect of tPA on the expression levels of Shh signaling components in BMECs. As shown in Fig. 5, the protein and mRNA levels of Shh, Smo, Ptch1, Gli-1, Gli-2, and Gli-3 were significantly increased in BMECs after OGD or Shh administration, but significantly decreased with tPA treatment. The protein and mRNA levels of ZO-1 and claudin-3 in BMECs subjected to OGD were lower than those in normal BMECs, but increased significantly after Shh administration.

## Discussion

The BBB is composed of endothelial cells, pericytes, and the end-feet of astrocytes. Among these, the intact tight junctions of brain capillary cells are critical for normal blood–brain barrier function, so that the endothelial cell barrier line is critical for preventing toxic substances from entering the brain [25]. BMECs can be detected and used as a cellular index of BBB damage [26]. This study described BBB disruption in BMECs subjected to OGD, as evidenced by the decreased TEER of cells, and tPA administration promoted OGD-induced BBB damage. Furthermore, we demonstrated that administration of high-concentration tPA acted on the Shh signaling pathway in BMECs, inhibiting it to enhance OGD-induced cell damage.

tPA, which affects neurite outgrowth, is expressed in many types of neural cells in the developing brain, including astrocytes and BMECs [27, 28]. To date, tPA is the only approved therapy for stroke, whereby intravenous injection of the thrombolytic agent promotes early reperfusion [29]. However, tPA potentiates neuronal death both *in vitro* and *in vivo*, even when administered intravenously. Cerebral hemorrhage after tPA infusion is relatively common, and in rare cases, tPA infusion may result in fragmentation of a cardiac thrombus, leading to an ischemic cerebral stroke [30]. Accordingly, despite clear overall benefits from its fibrinolytic action, there exists a legitimate need to explain how exogenous tPA can contribute to brain damage. In the present study, the mechanism of tPA-induced ischemia reperfusion damage was explored using an OGD model established in BMECs. Results revealed that administration of 300 µg/mL tPA significantly enhanced OGD and induced BMEC injury. The TEER and proliferation of BMECs were decreased and the cell cycle was blocked at the G0–G1 phase. Furthermore, the Shh signaling pathway was inhibited in rat BMECs by tPA treatment, which decreased the protein levels of Shh, Smo, Ptch1, Gli-1, Gli-2, and Gli-3.

Shh, as a mitogen and morphogen, is involved in vascular proliferation, differentiation, and maturation [31]. Recent researches have shown that the elements of the Shh signaling pathway are increased after ischemia in some tissues, including brain and myocardium [32, 33]. Moreover, blocked Shh signaling aggregates the level of brain edema in ischemic stroke [34]. However, whether Shh is involved in tPA-related intracerebral hemorrhage and BBB disruption during thrombolytic reperfusion remains unclear. In this study, we found that tPA-enhanced OGD-induced BMEC damage was eliminated by treatment with Shh, and the effects were reversed by cyclopamine. Cyclopamine is an antagonist of Smo that specifically inhibits the Shh pathway [35]. The Shh signaling pathway regulates apoptosis through the Smo protein in endothelial cells, and Shh administration accelerates wound healing by enhancing endothelial progenitor cell-mediated microvascular remodeling [18, 36]. The results of the present study indicated that tPA decreased cell proliferation and permeability transition by decreasing the TEER in BMECs. The underlying mechanism was mediated, at least partially, through the regulation of the expression of Shh signaling pathway components.

Previous studies have suggested that tPA could diffuse in the brain parenchyma and its deleterious effects such as BBB disruption would counteract its beneficial thrombolytic action [37, 38]. Moreover, the Shh signaling pathway is considered to regulate BBB permeability by downregulating tight junction proteins in astrocytes [39]. The present study further suggests that Shh signaling provides a barrier-promoting effect and endogenous anti-inflammatory balance with respect to central nervous system-directed immune attacks against endothelial cells and perivascular astrocytes, which compose the BBB. Here, we first demonstrated that Shh was involved in tPA-induced reduction of TEER in BMECs. Inhibition of the Shh signaling pathway in BMECs might contribute to the tPA-induced disruption of the endothelial barrier and promote cell injury.

In conclusion, this study demonstrates the direct involvement of tPA in the process of OGD-induced injury to BMECs. Blockage of the Shh signaling pathway might be one of the mechanisms by which tPA induces ischemia reperfusion injury. The precise mechanism by which tPA enhances ischemic injury requires further investigation.
